# An Interesting Case of Asymptomatic Cholera in the Setting of Large Bowel Obstruction

**DOI:** 10.7759/cureus.52854

**Published:** 2024-01-24

**Authors:** Nida Ansari, Sacide S Ozgur, Noor Bittar, Gabriel Melki, Yasmeen Sultana

**Affiliations:** 1 Internal Medicine, St. Joseph's Regional Medical Center, Paterson, USA; 2 Internal Medicine, St. George's University School of Medicine, St. George's, GRD; 3 Gastroenterology, St. Joseph's Regional Medical Center, Paterson, USA; 4 Infectious Disease, St. Joseph's Regional Medical Center, Paterson, USA

**Keywords:** vibrio cholerae, non-endemic region, endemic infections, non-bloody diarrhea, cancer colon, large-bowel obstruction

## Abstract

Vibrio cholerae is the culprit behind many endemics globally. Classically characterized by profuse diarrhea with a “rice water” description, cholera can be fatal if not treated promptly. However, infected individuals can present with little to no symptoms. These individuals allow for a carrier state and play a large part in the survival of an endemic. Asymptomatic patients can present in areas where Cholera is not endemic. Herein, we present an atypical case of vibrio chloerae infection without diarrhea in the setting of large bowel obstruction secondary to colon cancer. We aim to highlight the unusual presentation of a cholera infection.

## Introduction

Vibrio cholerae has a prevalence of up to 1.3-4,000,000 cases worldwide, with a mortality rate of 21,000-140,000 [1]. However, of the reported cases, it has been found that only 5 to 10% are reported [1]. V. cholerae is a facultative, gram-negative, comma-shaped, oxidative-positive rod that is prevalent in endemic areas [2]. Fifty countries have been known to be endemic to V. cholerae; however, most are seen in Asian Africa, whose incidence is believed to be tied to the region's rainy season [2]. Two serotypes often implicated in outbreaks are 01 and 0139, with the former being more seen in recent outbreaks [2]. It is often found in food, typically shellfish, and in water that has been poorly sanitized [2]. Cholera is spread via the fecal-oral route, requiring a large dose for infectivity [2]. It leads to severe diarrhea due to cholera toxin, which binds to intestinal cells and activates adenylyl cyclase. This increases cyclic adenosine monophosphate (cAMP) levels, leading to excessive chloride secretion and reduced sodium absorption in the intestines [3]. The result is a significant water efflux into the intestinal lumen, causing watery diarrhea. If untreated, this rapid fluid loss can lead to severe dehydration and potentially be fatal [3]. Recognizing cholera infection when asymptomatic or masked symptoms due to other etiologies, such as bowel obstruction, is crucial to decrease outbreaks, morbidity, and mortality [3]. This case report presents a rare incidence of asymptomatic V. cholerae infection in a patient with colon cancer.

## Case presentation

A 52-year-old male with a history of hypertension, metastatic colon adenocarcinoma at the hepatic flexure with metastasis to the liver, and hepatic flexure stricture status post colonic stent, was admitted for five days of right-sided abdominal pain, constipation, nausea, and non-bloody, non-bilious emesis. He reported that his last bowel movement and flatus were the day prior. His bowel movement was slightly loose but not diarrhea. He denied diarrhea, melena, hematochezia, hematemesis, chills, fever, rash, chest pain, shortness of breath, or any further complaints. 

His colon cancer was diagnosed one year prior with a colonoscopy that showed adenocarcinoma of the ascending colon at the hepatic flexure, and subsequent right upper quadrant ultrasound and CT of the abdomen showed diffuse replacement of the liver with multiple metastatic nodules. He had been receiving folic acid, fluorouracil, and oxaliplatin (FOLFOX) chemotherapy and had completed his second cycle one month prior.

**Figure 1:**
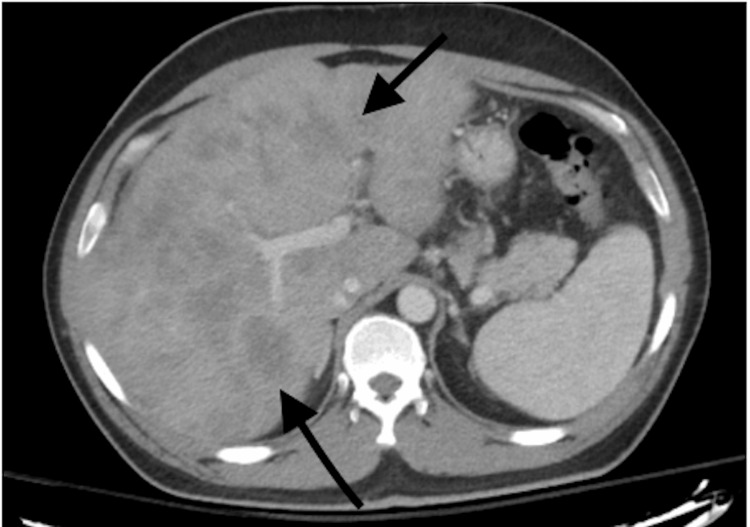
CT abdomen and pelvis with contrast, axial view, demonstrating diffuse liver metastases.

**Figure 2:**
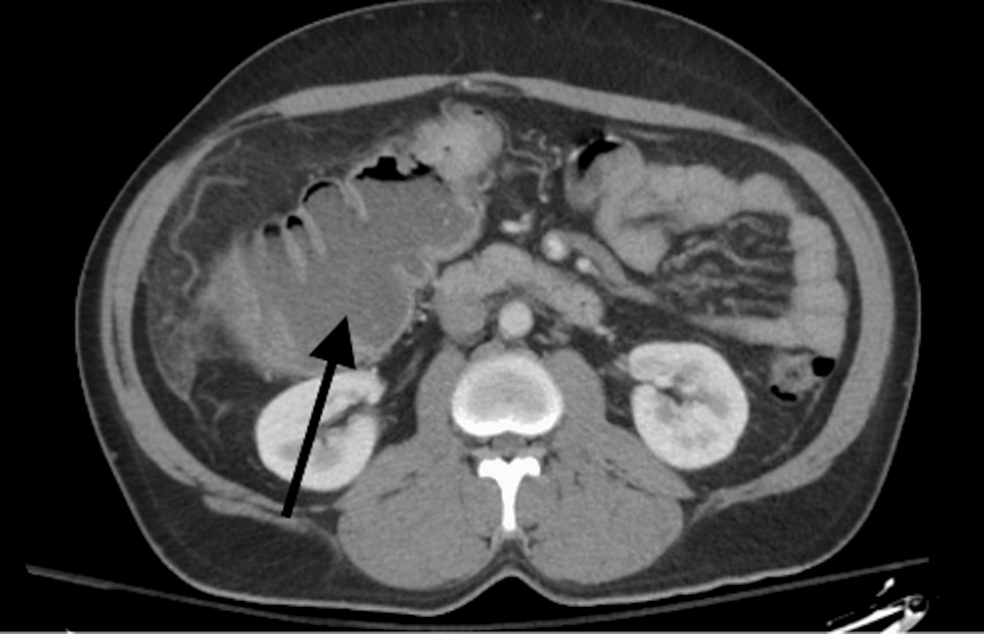
CT abdomen and pelvis with contrast, axial view, demonstrating large bowel obstruction in the transverse colon.

**Figure 3:**
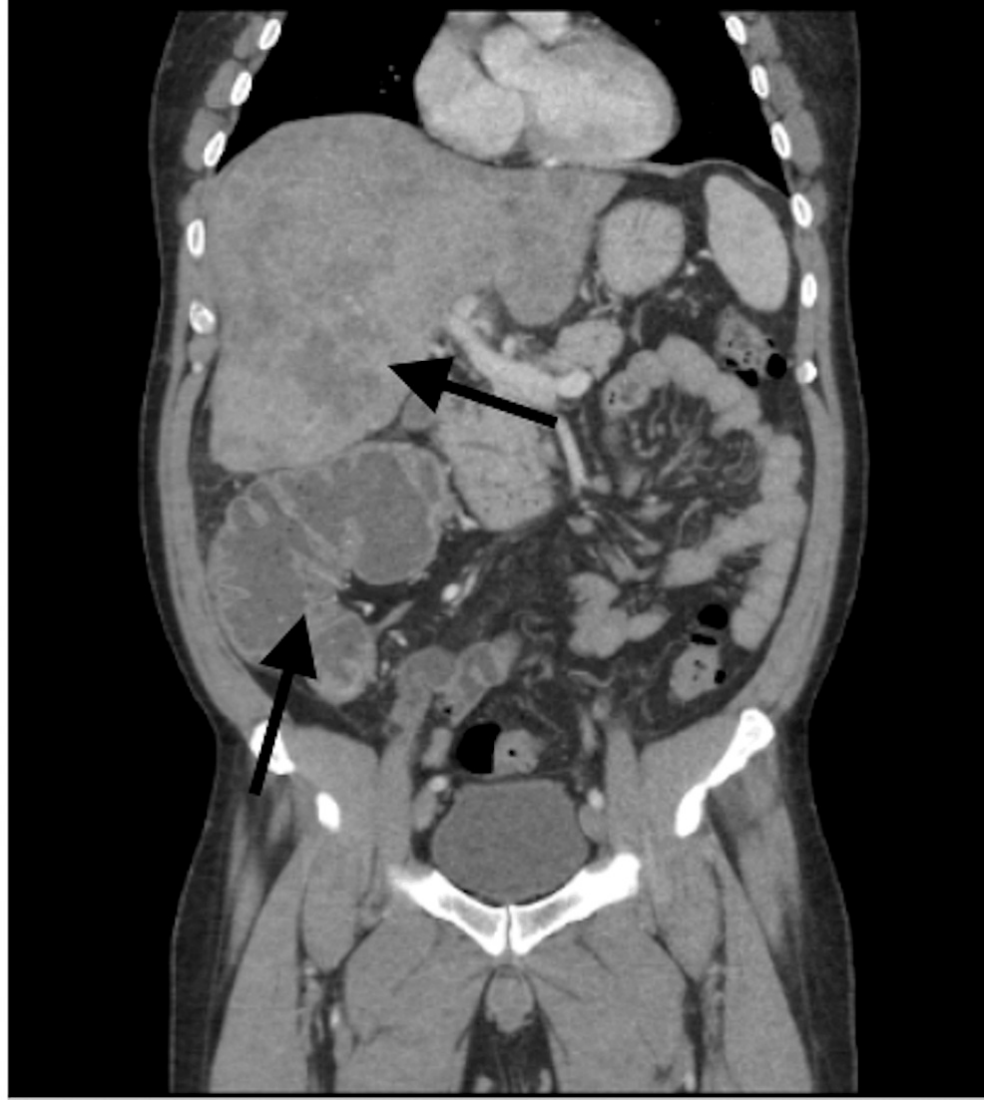
CT abdomen and pelvis with contrast, coronal view, demonstrating large bowel obstruction in the transverse colon and liver metastases.

His vital signs were unremarkable, and his physical exam was notable for diffuse abdominal tenderness and hepatosplenomegaly without guarding. A CT of the abdomen and pelvis without contrast showed large bowel obstruction at the level of the transverse colon with extensive hepatic metastatic disease with lesions up to 4.8 cm in size and evidence of colitis (Figures 1-3). CBC showed lymphopenia of 11.2/mm^3^. CMP showed calcium of 12.9 mg/dL, total bilirubin 1.8 mg/dL, alkaline phosphatase 631 units/L, ALT 87 units/L, and AST 87 units/L. Lipase was 4 units/L. Stool cultures were negative; however, the BioFire gastrointestinal polymerase chain reaction (PCR) panel of the stool was positive for E. coli (EAEC and EPEC) and V. cholerae.

The patient was diagnosed with non-diarrheal cholera in the setting of significant bowel obstruction secondary to colon cancer. Surgery was consulted for the patient’s lack of a bowel movement for one week associated with the abdominal pain and recommended colectomy with ostomy; however, surgical intervention would need to be delayed until the resolution of the patient’s vibrio infection. Infectious disease was also consulted, and the patient was started on 400 mg intravenous (IV) ciprofloxacin every 12 hours for five days. A repeat PCR panel six days later was negative, and the patient was able to proceed with the planned exploratory laparotomy with right colon mobilization and ileostomy creation, allowing for the resolution of his nausea and constipation. The patient’s chemotherapy was reinitiated one week postoperatively, but due to poor prognostic determination, he was discharged one month later on home hospice care.

## Discussion

Cholera colonizes in the small intestine, as the colon is not susceptible to the toxin [2]. Its toxin promotes increased secretion of electrolytes such as chloride, bicarbonate, sodium, and potassium, increasing osmotic forces and promoting diarrhea [2]. The toxin is noninvasive and confined only to the small intestine, so neutrophils are typically not seen in the stool [2]. 

Diagnosis can be made with clinical evaluation; however, laboratory testing can be done by acquiring stool samples [2,4]. When treating cholera, it is vital to ensure adequate fluid resuscitation [2]. Prompt treatment of fluid resuscitation can reduce mortality from over 10% to less than 0.5% [2,4]. Antibiotic therapy can be added once volume status is maintained [2]. Tetracyclines are the most implicated antibiotic; cautionary use should be noted in areas where resistance is known. For which macrolides or fluoroquinolone can be used [2].

Presentation of symptoms often varies; patients can be completely asymptomatic or as severe as profuse diarrhea [2,4]. The most common symptoms patients experience are diarrhea, abdominal discomfort, and vomiting [2]. Patients are typically also found to have rapid fluid and electrolyte loss; adults can experience up to 1 L/hour volume loss [2,4]. Diarrhea is often described as "rice water" consistency due to its mixing with bile and mucus [2]. Patients can experience symptoms of hypovolemia, such as decreased skin turgor, cool skin, and dry oral mucosa, and can experience poor perfusion of the body, resulting in lactic acidosis [2]. Additional electrolyte abnormalities such as hypokalemia and hypocalcemia can present, allowing for generalized muscle weakness and cramping [2].

To declare a patient symptomatic, the presence of diarrhea is often required [5]. However, whether patients are symptomatic or known, the condition of the inoculating dose the patients are exposed to [5]. However, the dose required to cause infection is unknown [5]. The incubation time could be inversely related to the inoculum dose [5]. Azman et al. stated that incubation can take up to 5 days with up to 7 days in the case of EL Toro Ogawa strains [5]. Another contribution that could be seen is that once V. chlorae is expelled from a human host, it enters a state of hyperinfectivity, possibly allowing for shorter incubation [5]. Previous exposure to cholera can possibly provide some form of protection [5]. Regardless, those who are transiently infected or asymptomatic largely contribute to the spread of infection [3,6]. Long-term carriers can exist but are exceedingly rare [3]. In the case of our patient, we cannot confidently establish whether the patient's presentation was secondary to asymptomatic infection or due to mechanical obstruction. However, this presentation in itself is interesting and highlights the importance of the carrier state of cholera infections.

## Conclusions

Vibrio cholerae infection typically presents with profuse diarrhea, vomiting, and abdominal pain. This disease carries a large risk of mortality, given the extremely dehydrated state that patients can experience in the subsequent complications. However, patients can also be asymptomatic or minimally symptomatic. Other complications, such as significant bowel obstruction, can mask the full presentation of cholera infection. This was possibly the case with our patient, and we aim to highlight the uniqueness of our patient's presentation.
